# Effect of Blood Contamination on Marginal Adaptation and Surface Microstructure of Mineral Trioxide Aggregate: A SEM Study

**DOI:** 10.5681/joddd.2013.025

**Published:** 2013-08-30

**Authors:** Amin Salem Milani, Saeed Rahimi, Mohammad Froughreyhani, Mahdi Vahid Pakdel

**Affiliations:** ^1^Dental and Periodontal Research Center, Tabriz University of Medical Sciences, Tabriz, Iran; ^2^Assistant Professor, Department of Endodontics, Faculty of Dentistry, Tabriz University of Medical Sciences, Tabriz, Iran; ^3^Professor, Department of Endodontics, Faculty of Dentistry, Tabriz University of Medical Sciences, Tabriz, Iran; ^4^Associate Professor, Department of Endodontics, Faculty of Dentistry, Tabriz University of Medical Sciences, Tabriz, Iran; ^5^Post-graduate Student, Department of Prosthodontics, Faculty of Dentistry, Tabriz University of Medical Sciences, Tabriz, Iran

**Keywords:** Blood contamination, marginal adaptation, microstructure, mineral trioxide aggregate, scanning electron microscopy

## Abstract

**Background and aims:**

In various clinical situations, mineral trioxide aggregate (MTA) may come into direct contact or even be mixed with blood. The aim of the present study was to evaluate the effect of exposure to blood on marginal adaptation and surface microstructure of MTA.

**Materials and methods:**

Thirty extracted human single-rooted teeth were used. Standard root canal treatment was carried out. Root-ends were resected, and retrocavities were prepared. The teeth were randomly divided into two groups (n = 15): in group 1, the internal surface of the cavities was coated with fresh blood. Then, the cavities were filled with MTA. The roots were immersed in molds containing fresh blood. In group 2, the aforementioned procedures were performed except that synthetic tissue fluid (STF) was used instead of blood. To assess the marginal adaptation, “gap perimeter” and “maximum gap width” were measured under scanning electron microscope. The surface microstructure was also examined. Independent samples t-test and Mann-Whitney U test were used to analyze the data.

**Results:**

Maximum gap width and gap perimeter in the blood-exposed group were significantly larger than those in the STF-exposed group (p < 0.01). In the blood-exposed group, the crystals tended to be more rounded and less angular compared with the STF-exposed group, and there was a general lack of needle-like crystals.

**Conclusion:**

Exposure to blood during setting has a negative effect on marginal adaptation of MTA, and blood-exposed MTA has a different surface microstructure compared to STF-exposed MTA.

## Introduction

Mineral trioxide aggregate (MTA) was introduced in 1993 as a root repair material.^1^ Further studies revealed various favorable properties of MTA, including low cytotoxicity, excellent biocompatibility and sealing ability.^2-7^ It was gradually used in a variety of clinical situations such as root end filling, pulp capping, apexification of open apex teeth, repair of root resorption, as a coronal barrier, and even as a canal filling material.^8-12^ In these clinical situations, MTA may come into direct contact or even be mixed with blood during or after placement. In most of the studies on its properties, MTA was allowed to set in contact with distilled water, normal saline, or at 37ºC/100% relative humidity.^13-16^, These conditions do not properly mimic the clinical situation where MTA comes in contact with tissue fluids or blood after placement in perforation repair or as a retrofill material.

Some recent studies have shown that MTA may exhibit different properties in different environmental conditions.^17-21^, Sarkar et al^19^ attributed the favorable properties of MTA in clinical studies to the reaction of MTA with its environment and concluded that MTA is not an inert material; rather, it is bioactive. They showed that MTA, in contact with synthetic tissue fluid (STF), releases calcium ions, triggering the precipitation of hydroxyapatite on its surface. In that study, STF was used instead of distilled water to simulate the clinical situation. It was the first published study that showed the importance of environmental situation on the properties of MTA. Since then, many studies have used STF instead of normal saline or distilled water.^22-25^, However, as stated, MTA may be inadvertently mixed with blood during placement or come into contact with blood during setting. The question is whether contamination with blood influences the properties of MTA. To answer this question, some recent studies have evaluated the properties of MTA in contact with blood or serum.

In two separate studies, Nekoofar et al^20,26^ showed that MTA incorporated with blood has reduced compressive strength and microhardness, and concluded that in clinical situations in which blood becomes mixed with MTA, its physical properties are likely to be compromised. In another study, Vanderweele et al^27^ showed that contamination of perforation sites with blood before MTA placement significantly reduced resistance to displacement. However, some leakage studies have failed to show any influence of blood on sealing ability of MTA.^28,29^ Studies on marginal adaptation of various root-end filling materials have shown that MTA has the best adaptation to dentin.^30-32^ Shipper et al^33^ compared the marginal adaptation of MTA with amalgam and concluded that gaps were significantly smaller in MTA samples. The authors attributed better marginal adaptation of MTA to water absorption and expansion of MTA during hydration. However, these studies were carried out in the controlled laboratory conditions without any contamination. The present study was carried out to evaluate the effect of exposure to blood on marginal adaptation and surface microstructure of MTA.

## Materials and Methods

### Sample Selection and Preparation

Extracted human single-rooted teeth were used in this study. The teeth with incomplete roots, cracks, resorption, or caries were discarded. Thirty teeth were finally selected for the study. They were cleaned of attached tissues and stored in 4% formalin until use. The crowns were resected from the cement-enamel junction (CEJ) using a high-speed diamond bur under water spray. The canal length was measured by introducing an ISO #15 K-file into the canal until the tip was visible at the apex. Apical enlargement was carried out to ISO #40 K-file (Maillefer, Ballagius, Switzerland). The canals were flared up to ISO #60 (Maillefer, Ballagius, Switzerland) with step-back technique. Two milliliter of 2.5% sodium hypochlorite was used to irrigate the canals between files. The prepared canals were dried with paper points and filled with gutta-percha (Ariadent, Tehran, Iran) and AH26 sealer (Dentsply, Konstanz, Germany) using lateral compaction technique. The samples were incubated at 37˚C and 100% relative humidity for 48 hours. Then, the apical 3-mm of roots were resected perpendicular to the long axes of the teeth using a fissured diamond bur (Tizkavan, Tehran, Iran) in a high-speed handpiece. Root-end cavities were prepared to a depth of 3 mm using Kis-3D microsurgical ultrasonic (Spartan, Missouri, USA) with medium power and water spray. The prepared cavities were dried with paper points. The teeth were randomly divided into two groups (n = 15).

### Group 1 (Blood-exposed)

Human blood was obtained by phlebotomy from the first author. The root-end cavities were immediately filled with fresh blood and then gently aspirated.

This way, the internal surface of the canals was coated with blood before filling. The remaining fresh blood was immediately placed in a mold containing 50 IU of heparin (Alborzdarou, Tehran, Iran) per one milliliter of blood. MTA (AMTA; Angelus, Londrina, Brazil) was mixed with distilled water according to manufacturer’s instructions and incrementally placed into the cavity and compacted using prefitted Schilder pluggers (Dentsply Caulk, Milford, DE). The cavities were slightly overfilled, and the excess material was gently burnished. The roots were soaked in a vial containing heparin for a second, and immediately immersed in molds containing heparinized blood. The samples were incubated at 37˚C and 100% relative humidity for 48 hours.

### Group 2 (STF-exposed) 

The aforementioned procedures were performed except that STF was applied to the canal walls, and the samples were immersed in STF after MTA placement.

### Scanning Electron Microscopy (SEM) Analysis 

The specimens were retrieved from the molds, rinsed with distilled water, air-dried and stored at 37˚C for 72 hours. The samples were viewed under a stereomicroscope (Carl Zeiss, Germany) at ×20 magnification to verify the integrity of root apices and exclude the samples with dentinal cracks extending to the MTA-dentin interface before SEM examination.

The roots were then mounted on an aluminum stub; sputter-coated with gold and viewed under SEM (Cam Scan, MV2300, Czech Republic). The specimens with dentinal cracks extending to the MTA-dentin interface were excluded. To assess the marginal adaptation, the two following variables were measured by a blind observer at different magnifications:

- “Maximum gap width” as the maximum distance between MTA and cavity walls was measured directly at MTA-dentin interface at ×2000 magnification (µm).
- “Gap perimeter” as the ratio of the gap perimeter to the perimeter of retrocavity margin was measured at ×300 magnification and rated on a score of 1 to 4:

1. Less than or equal to ¼ of the cavity margin
2. More than ¼ but less than or equal to ½ of the cavity margin
3. More than ½ but less than or equal to ¾ of cavity margin
4. More than ¾ of cavity margin

To assess the surface microstructure, the specimens were evaluated and compared at ×180, ×250, ×1000 and ×2000 magnifications regarding porosity, size and shape of crystals, and presence of microchannels.

### Statistical Analysis

All analyses were performed using the SPSS version 16 (SPSS Inc., Chicago, IL, USA). Independent samples t-test was used to compare the maximum gap width between the two groups. Mann-Whitney U test was performed to compare the means of gap perimeter in the two groups. The significance level was set at p = 0.05.

## Results

Preliminary evaluation of the samples under a stereomicroscope showed that two samples from the STF-exposed group and three samples from the blood-exposed group had dentinal cracks which did not extend to MTA-dentin interface. Therefore, the samples were not excluded and were prepared along with the other samples for SEM analysis. Evaluation of the samples by SEM showed that four samples (27%) from each group had cracks extending to MTA-dentin interface, probably as a result of dehydration and increased vacuum during preparation. These defective samples were excluded.

Maximum gap width and gap perimeter in the blood-exposed group were significantly higher than those in the STF-exposed group (p < 0.01; Tables 1 and 2).

**Table 1:** Summary Statistics of “maximum gap width” in MTA-dentin interface in experimental groups

Group	Mean (µm)	Std. Deviation
Blood-exposed (n = 11)	19.8	10.1
STF-exposed (n = 11)	4.5	3.3

MTA: Mineral trioxide aggregate; STF: Synthetic tissue fluid

**Table 2:** “gap perimeter” scores in experimental groups

Group	Score
	1	2	3	4
Blood-exposed (n = 11)	1(9.1)	5(45.5)	4(36.4)	1(9.1)
STF-exposed (n = 11)	8(72.7)	2(18.2)	0(0)	1(9.1)

STF: Synthetic tissue fluid

**Figure 1:**
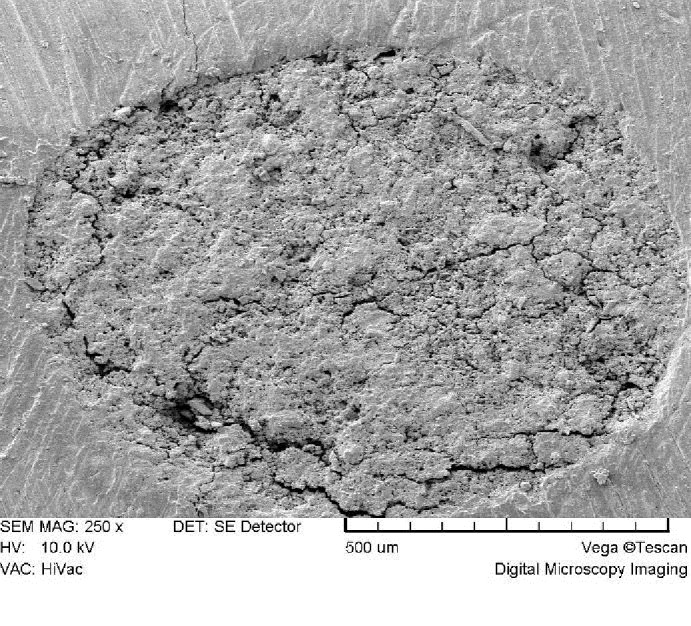
Porous rough surface of blood-exposed MTA (×250)

**Figure 2:**
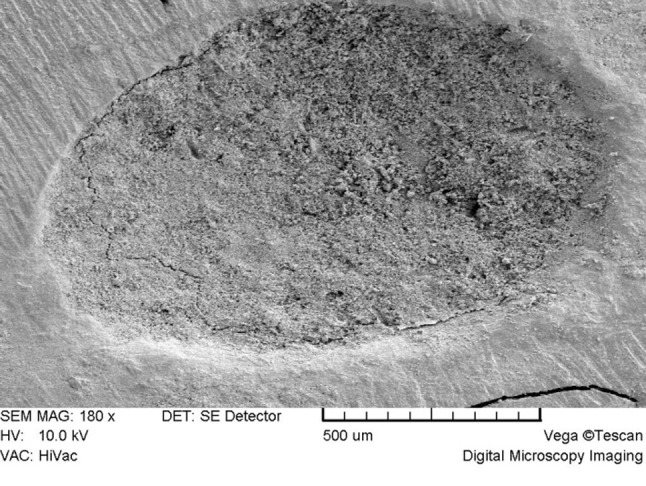
Porous rough surface of STF-exposed MTA (×180)

**Figure 3:**
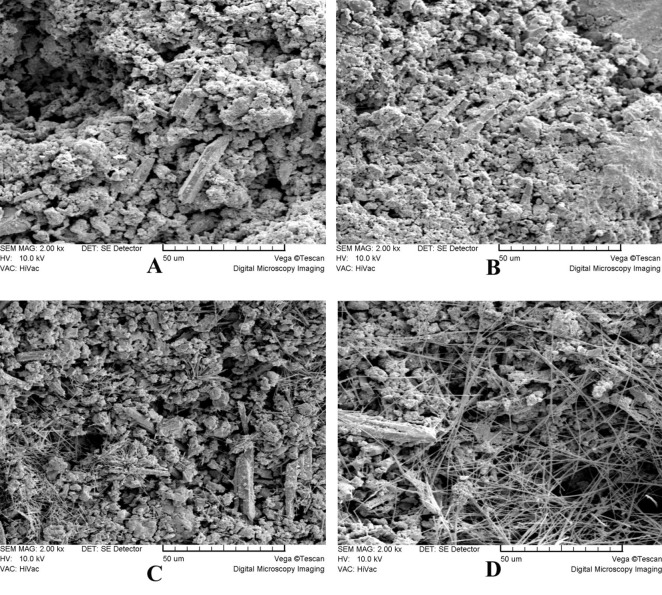
Surface microstructure of STF-exposed MTA (×2000). (A) Hexagonal, plate-like, and amorphous crystals are evident. (B) The crystals are covered by a poorly-crystalline superficial gel form structure in some area. (C) Small needle-like crystals are scattered between hexagonal and amorphous crystals in some areas. (D) Large needle-like crystals are evident in some areas.

**Figure 4:**
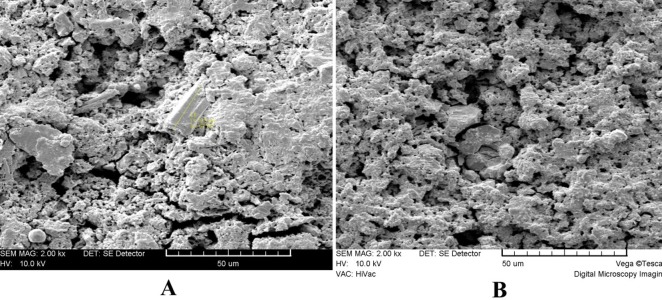
Surface microstructure of Blood-exposed MTA (×2000). (A) Numerous microchannels and few rounded hexagonal crystals partially covered by a gel form structure are evident. No needle like structures can be seen. (B) The crystals tend to be more rounded and partially covered by a poorly-crystalline structure.

In general, there were no distinct microstructural differences between the two groups at low magnifications (×100-×300). Both groups exhibited a porous rough surface (Figure 1 and 2). At higher magnifications (×1000-×2000), the STF-exposed group showed an irregular crystalline microstructure. It consisted of hexagonal and plate-like crystals with well-defined borders and amorphous crystals (Figure 3A) which were covered in some areas with a poorly-crystalline superficial gel-form structure (Figure 3B). Another finding was the presence of needle-like crystals which were distributed unevenly throughout the material. Some small needle-like crystals were scattered between hexagonal and amorphous crystals (Figure 3B); however, large needle-like crystals were

present in clusters in different parts of the material surface (Figure 3D). Blood-exposed MTA showed a relatively different microstructure at high magnifications. The microstructure consisted of a porous matrix with numerous microchannels (Figure 4) and fewer hexagonal crystals (Figure 4A) partially covered by a superficial poorly-crystalline gel-form structure (Figure 4A). The crystals tended to be more rounded and less angular compared with the STF-exposed group (Figure 4B), and there was a general lack of needle-like crystals.

## Discussion

The present study showed that exposure to blood negatively affects marginal adaptation of MTA to dentin. However, various leakage studies have shown similar sealing ability of blood-exposed and saline- or distilled water-exposed MTA.^28,29^ There is no published study on the comparison of the sealing ability of STF-exposed versus blood-exposed MTA. When MTA comes into contact with STF, calcium ions released by MTA react with the phosphate in STF leading to precipitation of an interfacial layer in the MTA-dentin interface.^19^ Earlier studies had proposed that the precipitate was hydroxyapatite.^18,19^ However, further studies showed that the precipitate mostly consisted of amorphous calcium phosphate which acts as a precursor to the formation of carbonate apatite which is the mineral phase of hard tissues.^34,35^ The ability of MTA to precipitate carbonate apatite in contact with STF in the MTA-dentin interfacial area and within dentinal tubules has been proposed as a mechanism of decreasing leakage,^24^ which may explain higher sealing ability of STF-exposed MTA in comparison with saline- or distilled water-exposed MTA.^23^ Studies have shown evidence of precipitation even within the first hour after immersion in STF;^24^ however, complete precipitation over the surface of MTA and in the MTA-dentin interface has been shown in the long term.^19,24,25^ In the present study, better marginal adaptation of STF-exposed MTA may be attributed to the precipitation of carbonate apatite. It is worth mentioning that better marginal adaptation of STF-exposed MTA does not mean less leakage. The sealing ability of blood- versus STF-exposed MTA needs further investigation.

Another finding of the present study was the different surface microstructure of MTA in contact with blood or STF. There is still no quantitative criterion to compare the groups regarding surface topography. Therefore, we used a descriptive approach in our study. The results showed that blood contamination caused fewer hexagonal crystal formation and general lack of needle-like ones. The crystals in MTA-exposed group tended to be more rounded and less angular compared with the STF-exposed group. These results were similar to those reported in the studies on the effect of acidic environment on microstructure of MTA.^36,37^ We have no explanation for the similarity of these findings. Some studies have tried to find a relationship between different surface microstructure of MTA and its properties. For instance, Nekoofar et al^26^ proposed that the absence of the needle-like crystals may have an adverse effect on surface microhardness. However, the direct relation of surface microstructure and different properties of MTA needs further investigations.

The results of the present study showed that MTA exposed to STF during setting may demonstrate different properties compared with blood-exposed MTA, which may overestimate the favorable properties of MTA in real clinical situations where MTA comes into contact with blood during setting. Therefore, contact with blood during the setting reaction is recommended in laboratory studies on the properties of MTA.

## Conclusion

Within the limitations of this in vitro study, it can be concluded that exposure to blood during setting has a negative effect on marginal adaptation of MTA, and blood-exposed MTA has a different surface microstructure compared to STF-exposed MTA.
